# Huddling substates in mice facilitate dynamic changes in body temperature and are modulated by *Shank3b* and *Trpm8* mutation

**DOI:** 10.21203/rs.3.rs-3904829/v1

**Published:** 2024-06-26

**Authors:** Jason G. Landen, Morgane Vandendoren, Samantha Killmer, Nicole L. Bedford, Adam C. Nelson

**Affiliations:** 1) Department of Zoology and Physiology, University of Wyoming, Laramie, WY, USA.; 2) University of Wyoming Sensory Biology Center, Laramie, WY, USA.

## Abstract

Social thermoregulation is a means of maintaining homeostatic body temperature. While adult mice are a model organism for studying both social behavior and energy regulation, the relationship between huddling and core body temperature (Tb) is poorly understood. Here, we develop a behavioral paradigm and computational tools to identify active-huddling and quiescent-huddling as distinct thermal substates. We find that huddling is an effective thermoregulatory strategy in female but not male groups. At 23°C (room temperature), but not 30°C (near thermoneutrality), huddling facilitates large reductions in Tb and Tb-variance. Notably, active-huddling is associated with bidirectional changes in Tb, depending on its proximity to bouts of quiescent-huddling. Further, group-housed animals lacking the synaptic scaffolding gene *Shank3b* have hyperthermic Tb and spend less time huddling. In contrast, individuals lacking the cold-sensing gene *Trpm8* have hypothermic Tb – a deficit that is rescued by increased huddling time. These results reveal how huddling behavior facilitates acute adjustments of Tb in a state-dependent manner.

## Introduction

Huddling – an active and close aggregation of animals – can serve multiple functions, from thermoregulation to social reward. Thermoregulatory huddling is widespread among animals and is hypothesized to provide an effective means of regulating core body temperature (Tb) and conserving energy. Huddling is cooperative in the sense that individuals bear the costs of donating body heat, but share the benefits^1^. Huddling can alter Tb by increasing the ambient temperature surrounding individuals in close contact, by reducing surface area to volume ratio and therefore heat loss, or by augmenting insulation^2,3^. Notably, most endotherms with the ability to huddle maintain a higher and more stable Tb than their isolated counterparts^2^. However, depending on the species, developmental stage, and environmental conditions, huddling can operate in conjunction with thermoeffector pathways to increase or decrease Tb to maintain a “set point” specific to a particular behavioral state^2,4^. For example, in adult penguins^5^ huddling results in lower Tb and reduced metabolic heat production, allowing individuals to maximize energy savings. Similarly, human babies in physical contact with their mother’s skin display reduced heat loss and metabolic output^6–8^. In contrast, huddling in rabbit pups results in higher Tb as well as thermoregulatory energy savings that can be channeled into competitive ability^9^.

For social animals, the close physical contact experienced during huddling can also reduce stress or be intrinsically rewarding. For example, individuals often display preferences for social contact or for contexts in which they previously experienced social contact^10,11^. Similarly, social contact can buffer the effects of stress^12–14^. In contrast, isolation and removal from physical contact with conspecifics can be aversive^15^ and have long lasting consequences^16^. For example, chronically isolated male mice display a reduction of huddle formation, but increased approach behavior, when re-introduced to a group setting^17^.

The house mouse, a model organism for studies of energy homeostasis and social behavior alike, displays extensive huddling behavior in the wild^18^ and in the laboratory^19,20^. While room temperature (RT, ~21°C) is thermoneutral (i.e., an ambient temperature where metabolic rate is at a minimum) for humans, it is well below thermoneutrality for mice, largely due to their high surface area to volume ratio^21–23^. This is especially apparent in mouse pups, which are born with immature capacity for thermoregulation^24–26^, and sex-specific thermoregulatory huddling strategies across the first eight days of postnatal development have been characterized^3,27^. In adult mice, individual housing at RT requires about a third of the energy budget to be devoted to cold-induced thermogenesis^28^. In contrast, group-housed mice at RT display lower thermal conductance (i.e., less heat loss) than individually-housed mice, and this effect is thought to be due to the energy-saving benefits of huddling^29^. In support of this idea, group-housed animals huddle more at lower temperatures^19^ and have overall lower metabolic rates than individually-housed mice^20^.

Despite the association between group housing and energy savings, the precise details of how huddling affects body temperature over the course of the day in adult mice are poorly understood^30^. For example, mice appear to engage in huddling both when awake and when asleep. While awake and sleep states in isolation are proposed to comprise regulated defenses of upper and lower Tb set points, respectively^4^, it is unclear how awake and asleep huddling states map onto these defended set points. Moreover, it is presently unclear how adult thermal states associated with huddling are affected by ambient temperature, sex, and/or genetic factors. One barrier to a more complete understanding of huddling behavior has been the difficulty of automating classification of behavior in a group setting. However, the development of computational tools to classify group-level behaviors will help disentangle the thermoregulatory and social components of huddling. We address these knowledge gaps.

Here, we develop a behavioral paradigm and computational tools to identify huddling-associated thermal states and how they are affected by internal (e.g., sex, age) and external (e.g., housing density, ambient temperature) factors in adult laboratory mice. We also investigated candidate genes expected to influence social interaction and thermosensation. First, *Shank3b* encodes a post-synaptic scaffolding protein, and mutations in this gene are associated with autism spectrum disorder (ASD), Phelan-McDermid syndrome^31^, and deficits in social interaction, including in *Shank3b*^−/−^ mouse models^32,33^. Second, the transient receptor potential channels *Trpv1* and *Trpm8* have established roles in thermosensation and thermoregulation. *Trpv1*, also known as the capsaicin receptor gene, is activated by hot stimuli^34^, and *Trpm8*, also known as the menthol receptor gene, is activated by cool stimuli^35^. Both *Trpv1*^36^ and *Trpm8*^37,38^ are involved in thermoregulation. We use animals with mutations in these genes to investigate their association with social thermoregulation.

Altogether, this study quantifies huddling behavior in over 300 48-hour recordings at the resolution of seconds to identify active and quiescent huddling substates that dynamically control body temperature, particularly in females. We show that huddling substates in group-housed animals are associated with lower Tbs than what are seen during bouts of inactivity in solo-housed mice. Intriguingly, the effect of active huddling can either increase or decrease core Tb, depending on the context. Finally, we show that the normal patterns of social thermoregulation in mice are fundamentally altered in *Shank3b* and *Trpm8* mutants.

## Results

### Body temperature is affected by housing density, sex, and ambient temperature.

**Figure 1:**
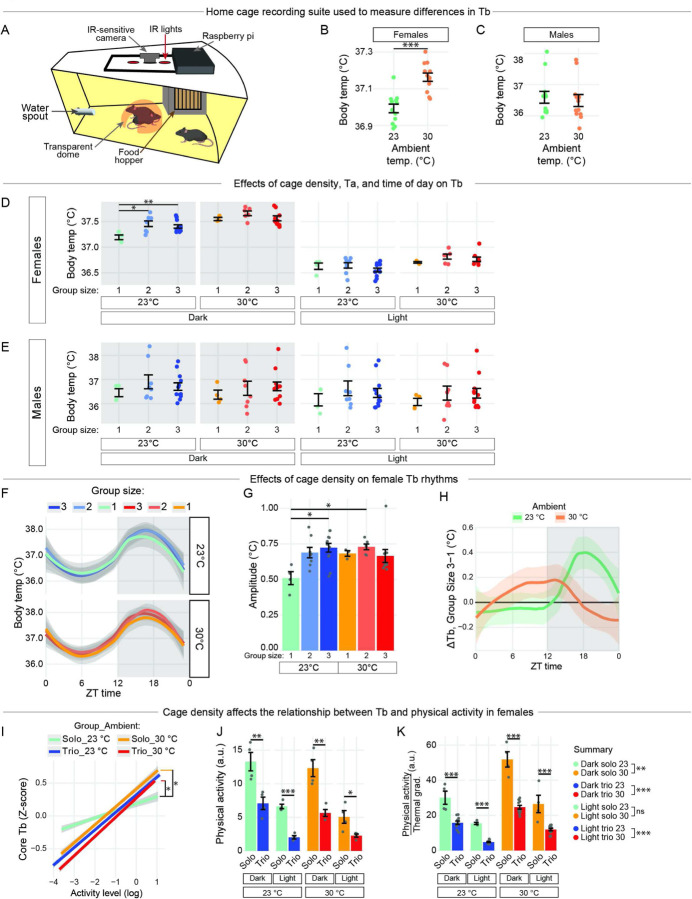
Body temperature is affected by sex, housing density, ambient temperature, and activity.

We set out to determine how thermal biology is affected by housing density. To do so, we developed a paradigm in which core body temperature (Tb) and activity of groups of one to three mice are recorded without any human interruption (Fig. 1A). The recordings occur over a 48-hour period with a 12/12 light/dark cycle in two different ambient temperatures (Ta): a standard vivarium temperature (Ta = 23°C)^39^ and a temperature near the lower critical temperature of the thermoneutral zone for adult male and female C57 mice (Ta = 30°C)^21,23,29,40–42^. Temperature loggers implanted in the abdomen of each mouse recorded Tb once per minute.

First, we found that female Tb was higher when housed at 30°C than at 23°C (Fig. 1B), whereas male Tb was not affected by ambient temperature (Fig. 1C). At 23°C, solo-housed females had significantly lower mean Tb compared to pair- or trio-housed females, but only during the dark cycle (Fig. 1D). In contrast, housing density had no effect on male Tb (Fig. 1E). These results suggest that group size affects female Tb below thermoneutrality, and are consistent with reports that mouse Tb is lower at 23°C than 30°C^2,23^, and that solo-housed animals have lower Tb compared to group-housed animals at 23°C^29^. In our system, however, male Tb was unaffected by housing density and ambient temperature. We therefore focused our next set of analyses on female mice.

To determine the effect of housing density on diurnal rhythms of female Tb, we performed cosinor analysis (Fig. 1F; SOM methods). Analysis of Tb over time revealed that 23°C solo-housed animals had decreased Tb amplitude (Fig. 1G), likely due to an inability to achieve peak Tb during the dark cycle. In males, no effects of group size or ambient temperature were observed. To account for inter-individual variation in Tb, we next calculated the difference in diurnal Tb when females were trio-housed vs. solo-housed, on a per-individual basis. At 23°C, females were on average cooler during the inactive period and warmer during the active period when trio-housed vs. solo-housed. However, at 30°C, this patten was abolished and partially reversed: females were warmer during the inactive period when trio-housed vs. solo-housed (Fig. 1H). These results suggest that housing density affects female Tb rhythms in an ambient temperature-dependent fashion, with the span of Tb reduced during solo housing. Our findings corroborate previous reports that solo-housed animals have an overall lower Tb due to increased heat loss^2,29^.

Because physical activity and Tb are related, and because physical activity may differ between social contexts, we next investigated the relationship between Tb and physical activity according to both housing density and ambient temperature using regression analysis. We measured physical activity in female mice using frame-to-frame pixel changes in the 48-hr video recording. The slope between activity level and Tb in 23°C solo-housed females was significantly weaker than both 23°C trio-housed mice and mice housed at 30°C, regardless of housing density (Fig. 1I). Thus, solo-housed females have a dampened relationship between physical activity and Tb, likely due to excessive heat loss; moreover trio-housed animals maintain an equivalent thermal profile at 23 and 30°C.

We next determined how cumulative activity levels were affected by housing density and ambient temperature. Solo-housed animals had significantly higher activity levels than trio-housed animals across both ambient temperatures and light/dark conditions (Fig. 1J). These results suggest that the lower Tb in 23°C solo-housed females (Fig. 1D) cannot be explained by a decrease in physical activity. To further examine the relationship between physical activity, ambient temperature, and Tb, we analyzed an $\frac{\text{Actitvity}}{\text{Thermal}\;\text{gradient}}$ quotient, where thermal gradient is defined as [𝑇_*b*_ − 𝑇*ambient*]. Because, under most conditions, physical activity is tightly correlated with total energy expenditure^28,42^, this metric is related to thermal conductance. (i.e., $\frac{\text{Total}\;\text{energy}\;\text{expenditure}}{\text{Thermal}\;\text{gradient}}$). Here, we found that solo-housed animals had a greater activity/thermal gradient quotient than their trio-housed counterparts, a result driven by both lower Tb and higher activity (Fig. 1K). Thus, solo-housed animals may have a reduced ability to conserve heat during inactive periods of the day, particularly at 23°C. Together, these results suggest that group size and ambient temperature play important roles in determining the thermal profiles of laboratory mice.

### Huddling behavior is affected by sex, light/dark cycle, and ambient temperature.

**Figure 2:**
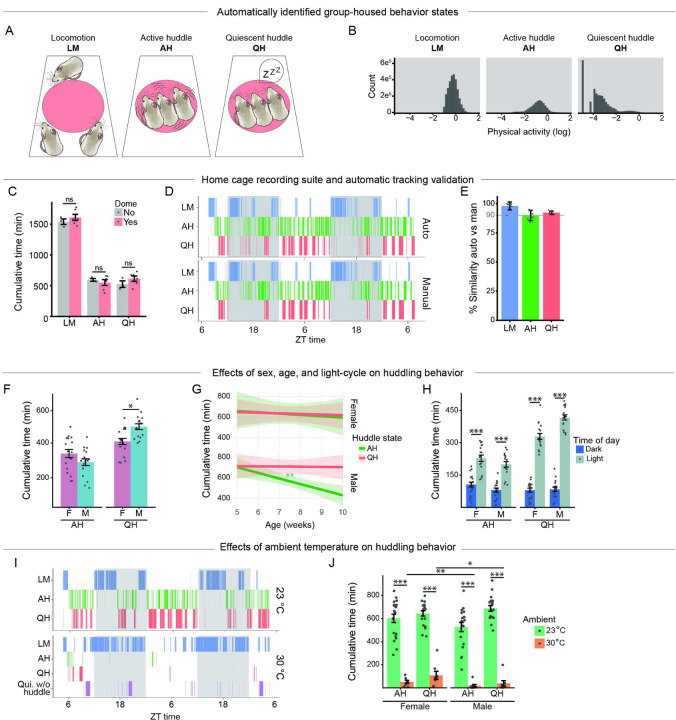
Huddling behavior is affected by sex, light/dark cycle, and ambient temperature.

We next addressed how huddling behavior might contribute to our observation that housing density alters the thermal biology of mice. To do so, we used the home cage recording suite to monitor aspects of behavior in groups of three mice for 48 hours (Fig. 1A). For group-housed mice, we defined three behavioral states: 1) locomotion (LM; group members all display high levels of physical activity), 2) active huddle (AH; all group members are huddling while displaying some physical activity), and 3) quiescent huddle (QH; all group members are huddling, but not displaying physical activity). Active and quiescent huddling substates were defined by direct physical contact between group members. Together, behavioral states were determined by a combination of location, physical contact, and activity level (Fig. 2A–B). The IR-transparent dome hut placed in the cage helps consolidate huddling to a single location, improving analysis performance without impact on the quantity of huddling (Fig. 2C). This surveillance-style recording suite, in conjunction with an automated analysis pipeline, quantifies huddling substates with approximately 90% accuracy compared to manual scoring (Fig. 2D–E). Thus, the home cage recording suite is a novel system for automated analysis of huddling substates over 48-hr time periods.

Using this paradigm, we quantified how huddling behavior changes due to different internal (e.g., sex, age) and external (e.g., light/dark cycle, ambient temperature) factors in trios of mice. For males and females from five to 10 weeks of age at 23°C, the cumulative time spent active huddling was approximately five hrs (300 min), and cumulative time spent quiescent huddling was approximately 7.5 hrs (450 min). While males and females spent an equivalent time active huddling, males spent more cumulative time quiescent huddling (Fig. 2F). From five to 10 weeks of age, males gradually reduced the amount of time spent active huddling, while time spent quiescent huddling was stable across this period. In contrast, females spent an equivalent and stable amount of time active and quiescent huddling across this period (Fig. 2G). In the huddling ethogram, time spent active and quiescent huddling appears to be time-of-day dependent (Fig. 2D). We therefore quantified the effect of the light/dark cycle on huddling. Although readily detectable throughout the 24-hr period, active and quiescent huddling substates were more common during the light cycle compared to the dark cycle in males and females (Fig. 2H). Thus, huddling behaviors are dependent on age, sex, and time of day.

The physical contact experienced during huddling can be considered either a thermoregulatory behavior ^1,2,9^ or a rewarding social behavior ^43–46^. The adult mouse thermoneutral zone (approximately 29–33°C^21,23,29,40–42^) is well above the standard ambient temperature of animal vivaria^39^, and the drive to huddle for thermoregulatory benefit may be particularly strong at 23°C. To determine whether mice are motivated to huddle for social reward in the absence of thermoregulatory need, we quantified cumulative huddling behavior in five- to 10-week-old mice at 30°C. Notably, huddling was nearly abolished in males and significantly reduced in females across the 48-hr period (Fig. 2I–J). Nevertheless, at 30°C, there were still periods when animals were quiescent but not making physical contact (i.e., “quiescent without huddling”) (Fig. 2I), a behavior rarely observed at 23°C (e.g., Fig. 2D), suggesting animals prefer to sleep alone at 30°C. While both males and females showed decreased time spent huddling at 30°C, females displayed significantly more time in AH and QH than males (Fig. 2J), suggesting a combination of social and thermoregulatory components of huddling in females. Together, these data indicate that huddling behavior is dependent on ambient temperature and, at standard room temperature, serves a primarily thermoregulatory function.

### Huddling among females facilitates an energy saving thermal profile at room temperature.

**Figure 3:**
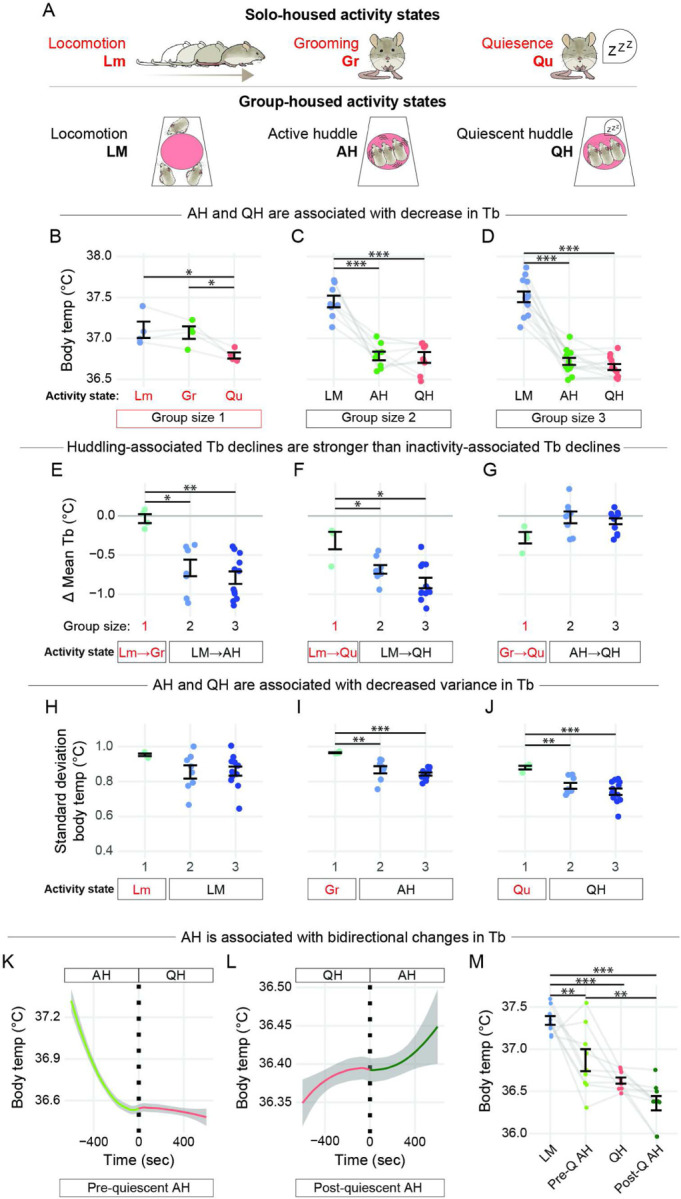
Huddling among females facilitates an energy saving thermal profile at room temperature.

Next, to directly link thermal and behavioral states in solo and group-housed animals, we monitored Tb and behavior over 48-hr periods using the home cage recording suite. Using housing rotations, we examined the same individuals either separated, with one sibling, or with two siblings during 48-hour recordings (SOM Methods). This design allowed us to control for between-individual variation while preventing long-term effects of isolation such as cold adaption^47^ and antisocial behavior ^48^. For solo-housed animals, we designated three different behavioral states corresponding to the behavioral states of group-housed animals: 1) locomotion (Lm; high levels of physical activity), 2) grooming (Gr; low levels of physical activity), and 3) quiescence (Qu; no physical activity) (Fig. 3A and S1A). Here, we aggregated data across the light/dark cycle to maximize the number of behavioral bouts in our linear mixed-effect modeling of Tb (SOM methods).

We compared mean Tb for each behavioral state in solo-, pair-, and trio-housed mice at ambient temperatures of both 23 and 30°C. In females, we observed a significant decrease in Tb from the highest to lowest behavioral state across all group sizes and ambient temperatures (Fig. 3B–D, S1B-D). These findings are consistent with other reports showing Tb is positively correlated with activity level^23,28,29,49^. However, patterns between solo and group-housed females were different. In solo females, quiescence was significantly lower than grooming and locomotion (Fig. 3B, S1B). In contrast, in group-housed females, both active and quiescent huddling were lower than locomotion (Fig. 3C–D). Thus, both huddling substates are associated with reduced Tb, even in ambient temperatures with minimal thermal stress (Fig. S1C-D).

To quantify the magnitude of change in Tb (**Δ**Tb), we compared mean **Δ**Tb between behavioral states in solo-housed animals (i.e., locomotion to grooming Lm→Gr, locomotion to quiescence Lm→Qu, and grooming to quiescence Gr→Qu), and equivalent behavioral state transitions in group-housed animals (i.e., locomotion to active huddle LM→AH, locomotion to quiescence huddle LM→QH, and active huddle to quiescence huddle AH→QH). We first examined **Δ**Tb at 23°C (Fig. 3E–G). For solo-housed animals, there was a negative **Δ**Tb between quiescence and both grooming and locomotion (Fig. 3F–G, light green points), confirming previous reports that sleep is associated with lower Tb^50–52^. In contrast, for group-housed animals, there was a strong negative **Δ**Tb between locomotion and both active and quiescent huddling (Fig. 3E–F, light and dark blue points), and these huddling-associated decreases in Tb were significantly larger than the decreases in Tb from locomotion to grooming and quiescence in solo-housed animals (Fig. 3E–F). These results suggest that, below the thermoneutral zone (i.e., 23°C), active and quiescent huddling in females facilitate reductions in Tb that are stronger than those during comparable behavioral transitions in solo females, consistent with an energy saving model of social thermoregulation.

We next compared **Δ**Tb between behavioral states at 30°C, where huddling is far less common (Fig. 2J). For group-housed females, active huddling was associated with a negative **Δ**Tb compared to locomotion, and this decrease was greater than the decrease in Tb during grooming compared to locomotion in solo-housed females (Fig. S1E). In contrast to the 23°C data, at 30°C the **Δ**Tb from locomotion to quiescence huddling was equivalent to the **Δ**Tb from locomotion to quiescence in solo animals (Fig. S1F). Finally, for solo-housed animals, the **Δ**Tb from grooming to quiescence resulted in a decrease in Tb that was significantly greater than the **Δ**Tb from active huddling to quiescent huddling (Fig. S1G). These results suggest that, at 23°C and 30°C, both active and quiescent huddling can promote drastic decreases in Tb.

Huddling substates could facilitate transitions to a Tb set-point for rest (regardless of housing density), or to a set-point unique to huddling. To address this, we examined the absolute Tb associated with different behaviors. At 23°C, locomotion-associated Tb was higher in group-housed compared to solo-housed females (Fig. S1H). In contrast, active huddling was associated with a lower Tb than solo grooming, and quiescent huddling in trios was associated with a lower Tb than solo quiescence (Fig. S1I-J). These trends were largely lost at 30°C (Fig. S1K-M). Thus, under conditions of cold-induced thermogenesis, although group-housed animals become warmer than solo animals during locomotion, huddling facilitates lower rest-associated Tbs. Because group-housed animals, compared to solo-housed animals, have higher Tb during the active phase (Fig. 1D and^29^), lower physical activity (Fig. 1J and^29^), and lower activity/thermal gradient quotient (Fig. 1K), these data suggest that both active and quiescent huddling facilitate long-lasting energy-savings.

In addition to lowering Tb, reducing short-term Tb variance may be another way to conserve energy^53^. This notion is based on the principle that fluctuations in body heating will require more work given a certain body mass and heat capacity. Accordingly, intra-individual variance in Tb (as measured by Tb standard deviation, Tb SD) was higher at 23°C compared to 30°C, where mice experience a reduced rate of heat transfer to the environment (Fig. S2A). We then evaluated mean Tb SD between solo and group housed females at comparable behavioral states. At 23°C, whereas Tb SD during locomotion was equivalent in solo- and group-housed females (Fig. 3H), Tb SD during active huddling and quiescent huddling were lower than grooming or quiescence in solo-housed animals, respectively (Fig. 3I–J). At 30°C these trends were diminished, although Tb SD during quiescent huddling was still lower than solo quiescence (Fig. S2B-D). These results suggest that huddling affords stabilization of Tb that is otherwise not available to solo-housed animals, and this stability is more apparent at 23°C than 30°C, consistent with the notion that huddling is an energy saving behavioral strategy.

### Huddling has weaker effects on male thermal profiles.

We next investigated social thermoregulation in males using the same approach (Fig. S3). While female Tb decreased from the highest to lowest activity state across all group sizes and ambient temperatures (Fig. 3B–D, S1B-D), these trends were weaker in males (S3B-C). This was especially evident when looking at the magnitude of change (**Δ**Tb). Group-housed females displayed stronger decreases in Tb from locomotion to huddling states than solo-housed females did from locomotion to inactive states (Fig. 3E–F, S1E-F). This pattern was not observed in males: decreases in Tb from active to huddling states in groups were equivalent to decreases from active to inactive states in solo-housed males (Fig. S3D-E left and middle panels). Next, we discovered that, compared to solo-housed females, group-housed females displayed higher Tb during locomotion, but lower Tb during quiescent states, (Fig. S1H-J), and this pattern was not observed in males: there were no differences in Tb comparing solo- and group-housed males for any behavior or ambient temperature (Fig. S3F-G). Finally, at 23°C, female Tb SD tends to decrease from locomotion states to quiescence, regardless of group size, whereas this effect is diminished at 30°C (Fig. S2B-D). However, this pattern is eliminated in males, where they show no effect of behavior on Tb SD in 23°C, and only a decrease in Tb SD for trios at 30°C comparing high to low activity states (Fig. S3H-I). Thus, although male groups display both active and quiescent huddling, these behaviors do not confer significant thermoregulatory changes as seen in female groups.

### Female active huddling facilitates bidirectional body temperature changes before and after quiescent huddling.

Group-housed animals active huddle more at 23°C compared to 30°C (Fig. 2J), and the temporal patterning of active huddling appears correlated with quiescent huddling in the 48-hr ethogram (Fig. 2D & 2I). These observations suggest that active huddling may be a motivated behavior to facilitate Tb changes leading into and out of the energy-saving quiescent huddling state. To understand how Tb is affected by transitions between active and quiescent huddling, we used a computational strategy to characterize active huddling at the borders of quiescent huddling. We organized bouts of huddling into continuous “epochs” that were sustained for at least 900 frames, or three minutes. Next, we characterized active huddling epochs as either preceding a quiescent huddle (i.e., pre-QH active huddle) or following a quiescent huddle (i.e., post-QH active huddle), depending on whether they occurred within 10 minutes before the start, or 10 minutes after the end, of a quiescent huddle epoch (SOM methods). For these experiments, we focused on trios at 23°C, which exhibited strong active huddling associated decreases in Tb (Fig. 3D). Analysis of these huddling substates showed that during pre-QH active huddle epochs, Tb drops until approximately four minutes before the onset of QH and then stabilizes as the QH epoch begins (Fig. 3K). The opposite effect is seen in post-QH active huddle epochs, where Tb is stable coming out of the QH epoch but starts increasing about six minutes after the end of QH (Fig. 3L). These results suggest that active huddling operates in conjunction with physiological processes (e.g., vasomotor pathways or brown fat thermogenesis) to facilitate bidirectional changes in Tb, depending on whether it occurs before or after quiescent huddling.

We then examined mean Tb of each of these huddling substates. Consistent with our observation that Tb declines during huddling (Fig. 3D), we found that pre-QH active huddle, post-QH active huddle, and QH were all lower than the locomotion state (Fig. 3M). Moreover, mean Tb for active huddling was warmer during pre-QH compared to post-QH. An important consideration in the interpretation of these results is thermal inertia, where temperature loggers such as the ones used here can over-estimate Tb during cooling and under-estimate Tb during warming^54^. In light of this information, our results suggest that pre-QH active huddling is associated with a cooling transition that goes from a high to low Tb, while post-QH active huddling is a warming transition that goes from a low to high Tb. Because post-QH active huddling is the lowest observable Tb in our system (i.e., ~36.4°C, Fig. 3M), these data indicate that pre-QH active huddling may be a strategy to facilitate an energy saving state (and consequently heat loss) to reach the low defended Tb set-point of rest; similarly, at the low-point of this energy-saving state, post-QH active huddling may then be used to elevate Tb in preparation for the higher defended Tb set point of the active state^4^.

### *Shank3b* mutation is associated with decreased huddling and increased Tb.

**Figure 4:**
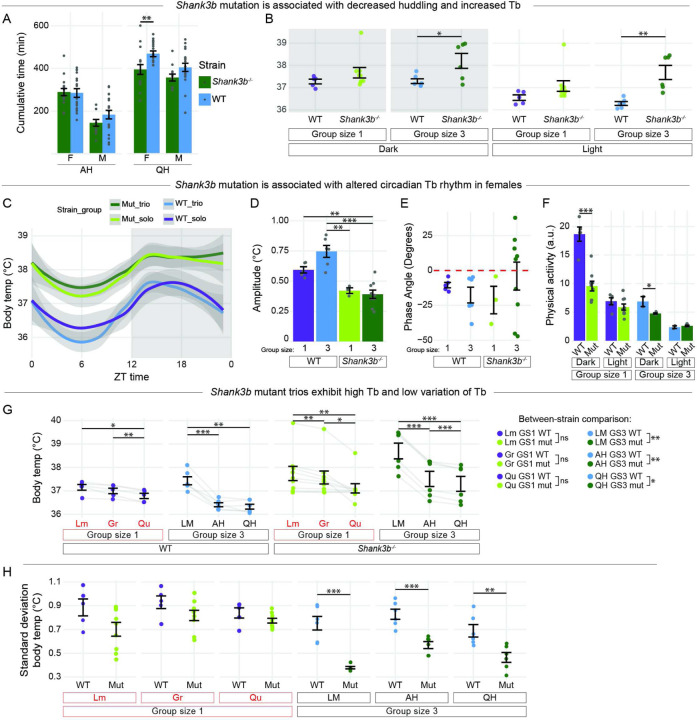
Shank3b^−/−^ mutation is associated with decreased huddling and increased Tb in females.

We next addressed how a genetic factor with an established role in prosocial interaction affects social thermoregulation at 23°C. The gene *Shank3b* encodes a post-synaptic scaffolding protein^55,56^, and, in humans, mutations in the gene are associated with autism spectrum disorder and Phelan-McDermid syndrome^57–59^. *Shank3b*^−/−^ mice show repetitive grooming behaviors and deficits in social interactions, particularly in the three-chamber sociability assay^33^. Here, we compared cumulative huddling time in *Shank3b*^−/−^ mutants^33^ and wildtype (WT) animals. Time spent active huddling was not affected by genotype. In contrast, mutant females spent less time quiescent huddling than WT females and, while males trended in the same direction, the difference was non-significant (Fig. 4A). These results suggest the antisocial effects described in *Shank3b*^*−/−*^ animals may generate a deficit in quiescent huddling, especially among females. We therefore focused all subsequent experiments on female mice.

We next analyzed the effect of *Shank3b* mutation on body temperature. Trio-housed mutants showed a significantly higher Tb during both light and dark cycles; solo-housed mutants showed a similar pattern, but the differences were non-significant (Fig. 4B). Thus, group-housed *Shank3b*^*−/−*^ animals spend less time quiescent huddling, and, unexpectedly, have a hyperthermic Tb compared to WT controls.

We then examined diurnal rhythms of Tb according to genotype. Mutant animals had on average higher mean Tb across all times of day (Fig. 4C). We further quantified these results by performing cosinor analyses and found that *Shank3b*^*−/−*^ animals had a lower Tb amplitude than their WT counterparts (Fig. 4D). Mutation did not affect phase angle, indicating that circadian Tb rhythm is dampened but not shifted (Fig. 4E). We next addressed whether high Tb in mutants was due to changes in physical activity. Surprisingly, solo-housed mutant animals had significantly less physical activity than solo-housed WT animals, while no differences in cumulative physical activity were measured in trio-housed animals (Fig. 4F). Together, these results suggest that *Shank3b* mutation affects circadian Tb rhythms by shifting them upwards and compressing them and that these Tb increases are not due to increased physical activity.

We next investigated the relationship between behavioral states and Tb in *Shank3b*^*−/−*^ animals. Like WT (Fig. 4G and Fig. 3B–D), mutant solo- and trio-housed animals showed Tb declines during grooming/quiescence and during active and quiescent huddling, respectively, although these declines were generally more significant than those observed in WT mice (Fig. 4G). Despite steeper Tb declines during huddling substates, group-housed *Shank3b*^*−/−*^ animals maintained higher Tb than wildtypes during all behavioral states, whereas solo-housed mutants showed no difference in Tb compared to WT in any behavioral states (Fig. 3G Between strain comparison). We next addressed the effect of *Shank3b* mutation on variance in Tb (Tb SD). Solo-housed animals displayed no effect of *Shank3b* on Tb SD for any behavioral state, whereas group-housed *Shank3b*^*−*/*−*^ animals showed a much lower SD Tb than wildtypes (Fig. 4H), suggesting that Tb may at a maximum.

Taken together, these results suggest that group housing induces hyperthermia in *Shank3b*^*−/−*^ females compared to WT. Although huddling can stabilize and reduce Tb in mutants, they do less of it, and it is insufficient to restore a normal Tb.

### Increased huddling in *Trpm8*^−/−^ mutants rescues hypothermic body temperature.

**Figure 5:**
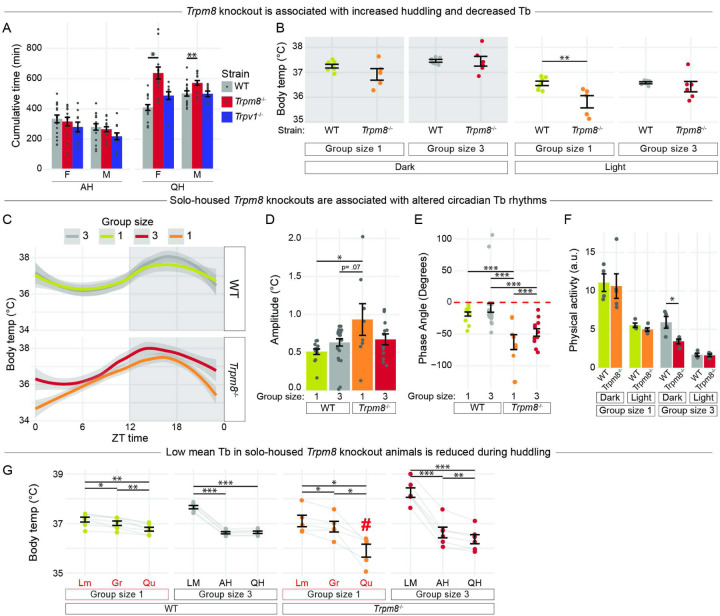
Increased huddling in Trpm8^−/−^ mutants rescues hypothermic body temperature in females.

We next addressed how two genetic factors with established roles in thermosensation affect social thermoregulation at 23°C. The cold-sensing menthol receptor *Trpm8* is activated at temperatures of approximately 26°C, with increasing activation as temperatures decrease to 8°C^60,61^. *Trmp8*^*−/−*^ mutants have disrupted thermosensation and thermoregulation and have lower Tb^62–64^. The warm-sensing capsaicin receptor *Trpv1* is activated at temperatures >43°C, a threshold similar to where heat evokes pain^61^. We compared huddling time in *Trpm8*^−/− 63^ and *Trpv1*^−/− 65^ mutant and homozygous WT animals. There was no effect of mutation on active huddling. In contrast, female and male *Trpm8*^−/−^ mutants showed a significant increase in quiescent huddling (Fig. 5A). These results suggest that the absence of *Trpm8*, but not *Trpv1*, results in altered huddling behavior at 23°C. Subsequent experiments therefore investigated *Trpm8*^−/−^ mutation in females, which display stronger thermoregulatory effects of huddling (Fig. 3).

We analyzed the effect of *Trpm8* mutation in solo- and trio-housed conditions. Trio-housed *Trpm8*^−/−^ and WT females showed equivalent Tb. In contrast, solo-housed *Trpm8*^−/−^ animals displayed a significant decrease in Tb during the light phase of the day (Fig. 5B). We then analyzed the effect of *Trpm8* mutation on diurnal rhythms of Tb and found that solo-housed *Trpm8*^−/−^ animals were notably different from WT (Fig. 5C). Cosinor analysis revealed that solo-housed *Trpm8*^−/−^ animals had a higher Tb amplitude compared to WT (Fig. 5D), indicating an increase in diurnal variation, and significantly lower phase angle than all other groups (Fig. 5E), indicating a left shift in their circadian Tb rhythm. These results suggest that solo-housed *Trpm8*^−/−^ females display both hypothermia and abnormal Tb rhythms compared to trio-housed *Trpm8*^−/−^ and WT females.

We next investigated the relationship between behavioral states and Tb in *Trpm8*^−/−^ animals. Compared to WT, trio-housed *Trpm8*^−/−^ animals exhibited decreased physical activity during the dark. In solo-housed animals, there was no effect of mutation on physical activity, indicating that reduced activity is not responsible for lower Tb in *Trpm8*^−/−^ mutants (Fig. 5F). Next, solo-housed WT females showed a steady decrease in Tb from the highest to lowest activity state (Fig. 5G). However, solo-housed *Trpm8*^−/−^ females displayed an abnormally low Tb during quiescence that was lower than all other conditions examined (Fig. 5G “#”, all p-values < .05). This was especially evident when looking at individual traces of Tb for solo-housed animals, where there were sudden drops in Tb to nearly 28°C (Fig. S4A-B), resembling bouts of torpor. These drastic drops in Tb were eliminated when the same animals were group-housed (Fig. S4C). Trio-housed WT and *Trpm8*^−/−^ females also showed similar trends of decreasing Tb at lower activity states (i.e., active and quiescent huddling). These results suggest that, compared to WT, *Trpm8*^−/−^ solo-housed females display abnormally low Tb during quiescence, but normal Tb during huddling when group housed.

We next addressed the effect of *Trpm8* mutation on variance in Tb (Tb SD). For solo-housed animals, wildtypes displayed a reduction in Tb SD during quiescence compared to grooming. In contrast, Tb SD in *Trpm8*^−/−^ solo-housed animals trended upwards during quiescence compared to grooming and locomotion (Fig. S4D), a pattern not observed in other experiments. For trio-housed animals, *Trpm8*^−/−^ again displayed an unusual trend of increasing Tb SD with lower levels of activity, although Tb SD during quiescent huddling was lower than that of active huddling (Fig. S4D).

Taken together, these results suggest that solo-housed *Trpm8*^−/−^ animals have a deficit in maintaining stable Tb and Tb rhythms, especially during quiescence. Because group-housed *Trpm8*^−/−^ animals exhibit increased huddling time and more normal Tbs, this deficit may be rescued by huddling.

## Discussion

Adult wild and laboratory rodents use huddling to thermoregulate^1,2,18,19^, but also as a form of social interaction^13,66–68^. Because laboratory mice are a model organism for the study of energy regulation and social behavior, there is a need to understand the precise details of how huddling affects body temperature (Tb). Here, we developed a system to quantify natural patterns of huddling behavior and Tb in the home cage of laboratory mice at the resolution of seconds. We identified active and quiescent huddling substates that are associated with distinct thermal profiles. Moreover, we found that these huddling substates are affected by group size, sex, ambient temperature, and the genes *Shank3b* and *Trpm8*.

### Active huddling in female groups facilitates dynamic changes in Tb at room temperature.

Our analysis of hundreds of 48-hr recordings revealed that huddling is a far more effective thermoregulatory strategy in female groups than in male groups. These findings extend previous reports that, in a cold environment, female pups maintain warmer surface temperatures and have more effective thermoregulatory huddling strategies than male pups^3,27^. We found that adult female Tb was lower at 23°C (i.e., below thermoneutrality) than at 30°C (i.e., near the thermoneutral zone). Moreover, solo-housed females at 23°C had lower Tb, decreased diurnal variability in Tb, and increased physical activity compared to group-housed females. These results are consistent with the observation that solo-housed females have greater thermal conductance and energy expenditure than their group-housed counterparts^29^. We then illuminated how this change in thermal biology was associated with huddling in group-housed females.

At 23°C, active and quiescent huddling in female groups, but not male groups, induced strong decreases in Tb (approximately −0.45°C). Notably, this led to a Tb that was lower than that of quiescence in solo-housed females. These huddling substates also resulted in a drastic reduction in Tb variance. In accordance with this observation, group-housed females, but not males, have lower total energy expenditure than their solo-housed counterparts at 23°C^29^. Because females huddled less at 30°C, our results suggest that active huddling at 23°C is primarily a motivated behavior to thermoregulate and save energy. Conversely, huddling among females at 30°C suggests possible social functions of this behavior.

Intriguingly, although active huddling in female groups was associated with lower Tb, it was associated with bidirectional changes in Tb. Specifically, active huddling epochs that came immediately before quiescent huddling displayed Tb decreases, whereas epochs immediately after quiescent huddling displayed Tb increases. This further suggests that active huddling is a motivated thermoregulatory behavior that, when aligned with other physiological processes (e.g., brown fat thermogenesis and cardiovascular pathways), facilitates significant modulation of Tb.

Taken together, our results suggest that sustained physical contact among females at 23°C triggers rapid thermoregulatory responses. This observation has important implications for the study of prosocial interactions because physical contact will result in heat exchange through conduction, increased insultion, and a reduction in the surface area to volume ratio of each individual^2,69^.

### *Shank3b* mutation causes hyperthermic Tb and decreased huddling in group-housed animals.

We investigated *Shank3b* on the premise that humans and mice with mutations in this gene show deficits in social behavior ^33,57,58^ and predicted that *Shank3b* mutants would huddle less than wildtypes. Indeed, *Shank3b*^−/−^ female, but not male, groups spent less time quiescent huddling than wildtypes. Surprisingly, despite being hypoactive, group-housed Shank3b^−/−^ females were characterized by low-variance, hyperthermic Tb compared to wildtypes. This result suggests that group-housed *Shank3b*^−/−^ females have disrupted thermal physiology and may near a Tb “ceiling”. The hyperthermia we observed in group-housed *Shank3b* mutants might indicate that these animals experience psychosocial stress associated with being housed together. Psychosocial stress is associated with elevated Tb in mice and humans^30,70–73^, and many neuropsychiatric disorders, including ASD, are linked to changes in efferent^30^ and afferent thermoregulatory pathways^74^. Considering *Shank3b* mutants are an animal model of ASD^31^, and that around half of individuals with ASD experience social anxiety^75–77^, it is possible that *Shank3b* mutants experience social stress-induced hyperthermic Tb.

Although huddling in group-housed *Shank3b*^−/−^ mice caused robust decreases in Tb and Tb-variance, these mutants spent less time quiescent huddling, and, as a result, less time in a low-Tb state. These results support the notion that *Shank3b*^−/−^ mice have social deficits but illustrate new associations with elevated Tb and impairments in huddling, suggesting that studies of rodent models of ASD should consider how thermoregulatory changes might interact with and contribute to social deficits. For example, mutation of the oxytocin gene in mouse pups results in reduced BAT thermogenesis and less cohesive huddling^78^.

### Hypothermia in solo-housed *Trpm8* mutants is rescued by the ability to huddle.

We investigated *Trpm8* and *Trpv1* on the premise that these genes are associated with thermosensation^62,63^ and thermoregulation^64^. Consistent with the observation that *Trpm8* deletion increases heat loss and reduces Tb^64^, we found that, compared to wildtypes, solo-housed *Trpm8*^−/−^ females displayed hypothermic Tb. Surprisingly, some animals even had Tb resembling torpor, with Tb reaching below 29°C, despite having *ad libitum* food. Intriguingly, these deficits were attenuated in a group-housed setting. Solo-housed *Trpm8*^−/−^ females had hypothermic Tb during quiescence, whereas group-housed *Trpm8*^−/−^ females had normal Tb during quiescent huddling. These observations suggest that housing density is an important consideration for studies of *Trpm8* mutants. Furthermore, investigation of social thermoregulation as a mechanism of coping with thermoregulatory dysfunctions in animal models is warranted.

### Conclusions.

Active and quiescent huddling substates at standard room temperature (23°C) are powerful and dynamic thermoregulatory behaviors for group-housed females. Studies of rodent social behavior are often conducted at room temperature, including studies of social homeostasis, or the ability of individuals to detect and regulate the quantity of social connections^79^. Our observation that mice are more likely to make physical contact at room temperature to thermoregulate suggests that both internal and external temperature should be an important experimental design consideration. Finally, our findings reveal mutations in *Shank3b* and *Trpm8* – two genes commonly used in studies of social interaction and energy balance, respectively – are associated with alterations in social thermoregulation. This study contributes to the idea that thermoregulation can be an important regulator of social interaction^78^.

## Limitations of the study

In this study, we examine how core body temperature (Tb) is associated with huddling substates in wildtype and mutant animals. Although we identify changes in Tb, our study does not test which thermoregulatory effector pathways (e.g., brown adipose fat thermogenesis and vasodilation) drive these changes. Although our logger implants can detect temperature changes at the resolution of one minute, our study did not address the precise effects of thermal inertia on the loggers at the resolution of seconds. One possible limitation of the study is that we arbitrarily set thresholds on activity level to define categorical behavioral states in group-housed and solo-housed animals. However, the fact that we could identify distinct thermal states for all of these states lends support to the notion that they are in fact distinct. Although we find that *Shank3b* mutation is associated with both a decrease in huddling and an increase in Tb when animals are group housed, our study does not investigate whether the increase in Tb is a driver of decreased huddling. Although this study used a longitudinal design to examine thermal profiles of the same individuals in different housing contexts with and without siblings, this required that animals were isolated for 72 hours at a time, which may have introduced physiological or behavioral changes. To mitigate these possible changes, mice were returned to their home cage for four days between experiments. Nevertheless, we did not determine how this cage rotation design affected the animals per se. Finally, it should be noted that for some experiments the sample size was rather low (e.g., 3 or 4 animals per experiment), and some conclusions could be due to insufficient statistical power.

## Data Availability

Raw data can be found on Mendeley. https://data.mendeley.com/preview/nwyfsn2bym?a=91f3a487-6543-490d-8176-444eaa9b0a8a

## References

[1] Haig D. (2008). Huddling: Brown Fat, Genomic ImprinFng and the Warm Inner Glow. Current Biology 18, R172–R174. 10.1016/j.cub.2007.12.040. 18302923

[2] Gilbert C., McCafferty D., Le Maho Y., MartreMe J., Giroud S., Blanc S., and Ancel A. (2010). One for all and all for one: the energeFc benefits of huddling in endotherms. Biological Reviews 85, 545–569. 10.1111/j.1469-185X.2009.00115.x. 20039866

[3] Harshaw C., and Alberts J.R. (2012). Group and individual regulaFon of physiology and behavior: A behavioral, thermographic, and acousFc study of mouse development. Physiology & Behavior 106, 670–682. 10.1016/j.physbeh.2012.05.002. 22580514 PMC3377822

[4] Škop V., Liu N., Xiao C., SFnson E., Chen K.Y., Hall K.D., Piaggi P., Gavrilova O., and Reitman M.L. (2024). Beyond day and night: The importance of ultradian rhythms in mouse physiology. Molecular Metabolism 84, 101946. 10.1016/j.molmet.2024.101946. 38657735 PMC11070603

[5] Gilbert C., Maho Y.L., Perret M., and Ancel A. (2007). Body temperature changes induced by huddling in breeding male emperor penguins. American Journal of Physiology-Regulatory, IntegraFve and ComparaFve Physiology 292, R176–R185. 10.1152/ajpregu.00912.2005. 16959865

[6] Asakura H. (2004). Fetal and Neonatal ThermoregulaFon. J Nippon Med Sch 71, 360–370. 10.1272/jnms.71.360. 15673956

[7] Fransson A.-L., Karlsson H., and Nilsson K. (2005). Temperature variaFon in newborn babies: importance of physical contact with the mother. Archives of Disease in Childhood - Fetal and Neonatal EdiFon 90, F500–F504. 10.1136/adc.2004.066589. PMC1721966 16244210

[8] Adamson K., Gandy G.M., and James L.S. (1965). The influence of thermal factors upon oxygen consumpFon of the newborn human infant. The Journal of Pediatrics 66, 495–508. 10.1016/S0022-3476(65)80114-7. 14264308

[9] Gilbert C., Blanc S., Giroud S., Trabalon M., Maho Y.L., Perret M., and Ancel A. (2007). Role of huddling on the energeFc of growth in a newborn altricial mammal. American Journal of Physiology-Regulatory, IntegraFve and ComparaFve Physiology 293, R867–R876. 10.1152/ajpregu.00081.2007. 17459914

[10] Dolen G., Darvishzadeh A., Huang K.W., and Malenka R.C. (2013). Social reward requires coordinated acFvity of nucleus accumbens oxytocin and serotonin. Nature 501, 179–184. 10.1038/nature12518. 24025838 PMC4091761

[11] Panksepp J.B., and Lahvis G.P. (2007). Social reward among juvenile mice. Genes Brain Behav 6, 661–671. 10.1111/j.1601-183X.2006.00295.x. 17212648 PMC2040181

[12] Denommé M.R., and Mason G.J. (2022). Social Buffering as a Tool for Improving Rodent Welfare. Journal of the American AssociaFon for Laboratory Animal Science 61, 5–14. 10.30802/AALAS-JAALAS-21-000006. PMC8786379 34915978

[13] Fukumitsu K., Kaneko M., Maruyama T., Yoshihara C., Huang A.J., McHugh T.J., Itohara S., Tanaka M., and Kuroda K.O. (2022). Amylin-Calcitonin receptor signaling in the medial preopFc area mediates affiliaFve social behaviors in female mice. Nat Commun 13, 709. 10.1038/s41467-022-28131-z. 35136064 PMC8825811

[14] Kikusui T., Winslow J.T., and Mori Y. (2006). Social buffering: relief from stress and anxiety. Philosophical TransacFons of the Royal Society B: Biological Sciences 361, 2215–2228. 10.1098/rstb.2006.1941. 17118934 PMC1764848

[15] Pietropaolo S, Feldon J, and Yee B.K. (2008). Nonphysical contact between cagemates alleviates the social isolaFon syndrome in C57BL/6 male mice. Behavioral Neuroscience 122, 505–515. 10.1037/0735-7044.122.3.505. 18513121

[16] Zelikowsky M., Hui M., Karigo T., Choe A., Yang B., Blanco M.R., Beadle K., Gradinaru V., Deverman B.E., and Anderson D.J. (2018). The NeuropepFde Tac2 Controls a Distributed Brain State Induced by Chronic Social IsolaFon Stress. Cell 173, 1265–1279.e19. 10.1016/j.cell.2018.03.037. 29775595 PMC5967263

[17] Endo N., Ujita W., Fujiwara M., Miyauchi H., Mishima H., Makino Y., Hashimoto L., Oyama H., Makinodan M., Nishi M., (2018). MulFple animal posiFoning system shows that socially-reared mice influence the social proximity of isolaFon-reared cagemates. CommunicaFons Biology 1, 225. 10.1038/s42003-018-0213-5. PMC6290015 30564746

[18] Peter Crowcrop (1966). Mice All Over (G. T. Foulis & Co., Ltd.).

[19] Batchelder P., Kinney R.O., Demlow L., and Lynch C.B. (1983). Effects of temperature and social interacFons on huddling behavior in Mus musculus. Physiology & Behavior 31, 97–102. 10.1016/0031-9384(83)90102-6. 6634982

[20] MarFn R.A., FiorenFni M., and Connors F. (1980). Social facilitaFon of reduced oxygen consumpFon in Mus musculus and Meriones unguiculatus. ComparaFve Biochemistry and Physiology Part A: Physiology 65, 519–522. 10.1016/0300-9629(80)90072-9.

[21] Gordon C.J. (2012). Thermal physiology of laboratory mice: Defining thermoneutrality. Journal of Thermal Biology 37, 654–685. 10.1016/j.jtherbio.2012.08.004.

[22] Gordon C.J. (2017). The mouse thermoregulatory system: Its impact on translaFng biomedical data to humans. Physiology & Behavior 179, 55–66. 10.1016/j.physbeh.2017.05.026. 28533176 PMC6196327

[23] Škop V., Guo J., Liu N., Xiao C., Hall K.D., Gavrilova O., and Reitman M.L. (2020). Mouse ThermoregulaFon: Introducing the Concept of the Thermoneutral Point. Cell Reports 31, 107501. 10.1016/j.celrep.2020.03.065. 32294435 PMC7243168

[24] Blumberg M.S. (2001). The Developmental Context of Thermal Homeostasis. In Developmental Psychobiology, Blass E. M., ed. (Springer US), pp. 199–228. 10.1007/978-1-4615-1209-7_6.

[25] Pembrey M.S. (1895). The Effect of VariaFons in External Temperature upon the Output of Carbonic Acid and the Temperature of Young Animals. The Journal of Physiology 18, 363–379. 10.1113/jphysiol.1895.sp000573. PMC1514592 16992261

[26] Robertson C.E., and McClelland G.B. (2019). Developmental delay in shivering limits thermogenic capacity in juvenile high-alFtude deer mice (Peromyscus maniculatus). Journal of Experimental Biology 222, jeb210963. 10.1242/jeb.210963. 31562187

[27] Harshaw C., Culligan J.J., and Alberts J.R. (2014). Sex Differences in Thermogenesis Structure Behavior and Contact within Huddles of Infant Mice. PLOS ONE 9, e87405. 10.1371/journal.pone.0087405. 24498099 PMC3909189

[28] Škop V., Guo J., Liu N., Xiao C., Hall K.D., Gavrilova O., and Reitman M.L. (2023). The metabolic cost of physical acFvity in mice using a physiology-based model of energy expenditure. Molecular Metabolism 71, 101699. 10.1016/j.molmet.2023.101699. 36858190 PMC10090438

[29] Škop V., Xiao C., Liu N., Gavrilova O., and Reitman M.L. (2021). The effects of housing density on mouse thermal physiology depend on sex and ambient temperature. Molecular Metabolism 53, 101332. 10.1016/j.molmet.2021.101332. 34478905 PMC8463779

[30] Rogers J.F., Vandendoren M., Prather J.F., Landen J.G., Bedford N.L., and Nelson A.C. (2024). Neural cell-types and circuits linking thermoregulaFon and social behavior. Neuroscience & Biobehavioral Reviews 161, 105667. 10.1016/j.neubiorev.2024.105667. PMC11163828 38599356

[31] Harony-Nicolas H., De Rubeis S., Kolevzon A., and Buxbaum J.D. (2015). Phelan McDermid Syndrome: From GeneFc Discoveries to Animal Models and Treatment. J Child Neurol 30, 1861–1870. 10.1177/0883073815600872. 26350728 PMC5321557

[32] Dhamne S.C., Silverman J.L., Super C.E., Lammers S.H.T., Hameed M.Q., Modi M.E., Copping N.A., Pride M.C., Smith D.G., Rotenberg A., (2017). Replicable in vivo physiological and behavioral phenotypes of the Shank3B null mutant mouse model of auFsm. Mol AuFsm 8, 26. 10.1186/s13229-017-0142-z. PMC5472997 28638591

[33] Peça J., Feliciano C., Ting J.T., Wang W., Wells M.F., Venkatraman T.N., Lascola C.D., Fu Z., and Feng G. (2011). Shank3 mutant mice display auFsFc-like behaviours and striatal dysfuncFon. Nature 472, 437–442. 10.1038/nature09965. 21423165 PMC3090611

[34] Mishra S.K., Tisel S.M., Orestes P., Bhangoo S.K., and Hoon M.A. (2011). TRPV1-lineage neurons are required for thermal sensaFon. The EMBO Journal 30, 582–593. 10.1038/emboj.2010.325. 21139565 PMC3034006

[35] Reid G. (2005). ThermoTRP channels and cold sensing: what are they really up to? Pflügers Archiv 451, 250–263. 10.1007/s00424-005-1437-z. 16075243

[36] Steiner Alexandre A., Turek Victoria F., Almeida Maria C., Burmeister Jeffrey J., Oliveira Daniela L., Roberts Jennifer L., Bannon Anthony W., Norman Mark H., Louis Jean-Claude, Treanor James J. S., (2007). Nonthermal AcFvaFon of Transient Receptor PotenFal Vanilloid-1 Channels in Abdominal Viscera Tonically Inhibits Autonomic Cold-Defense Effectors. J. Neurosci. 27, 7459. 10.1523/JNEUROSCI.1483-07.2007. 17626206 PMC6672610

[37] Reimúndez Alfonso, Fernández-Peña Carlos, García Guillermo, Fernández Rubén, Ordás Purificación, Gallego Rosalía, Pardo-Vazquez Jose L., Arce Victor, Viana Félix, and Señarís Rosa (2018). DeleFon of the Cold Thermoreceptor TRPM8 Increases Heat Loss and Food Intake Leading to Reduced Body Temperature and Obesity in Mice. J. Neurosci. 38, 3643. 10.1523/JNEUROSCI.3002-17.2018. 29530988 PMC6705917

[38] Almeida M. Camila, Hew-Butler Tamara, Soriano Renato N., Rao Sara, Wang Weiya, Wang Judy, Tamayo Nuria, Oliveira Daniela L., Nucci TaFane B., Aryal Prafulla, (2012). Pharmacological Blockade of the Cold Receptor TRPM8 AMenuates Autonomic and Behavioral Cold Defenses and Decreases Deep Body Temperature. J. Neurosci. 32, 2086. 10.1523/JNEUROSCI.5606-11.2012. 22323721 PMC3566779

[39] Speakman J.R., and Keijer J. (2013). Not so hot: OpFmal housing temperatures for mice to mimic the thermal environment of humans. Molecular Metabolism 2, 5–9. 10.1016/j.molmet.2012.10.002. PMC3757658 24024125

[40] Maloney S.K., Fuller A., Mitchell D., Gordon C., and Overton J.M. (2014). TranslaFng Animal Model Research: Does It MaMer That Our Rodents Are Cold? Physiology 29, 413–420. 10.1152/physiol.00029.2014. 25362635

[41] Reitman M.L. (2018). Of mice and men – environmental temperature, body temperature, and treatment of obesity. FEBS LeMers 592, 2098–2107. 10.1002/1873-3468.13070. 29697140

[42] Abreu-Vieira G., Xiao C., Gavrilova O., and Reitman M.L. (2015). IntegraFon of body temperature into the analysis of energy expenditure in the mouse. Molecular Metabolism 4, 461–470. 10.1016/j.molmet.2015.03.001. 26042200 PMC4443293

[43] Beery A.K., Lopez S.A., Blandino K.L., Lee N.S., and Bourdon N.S. (2021). Social selecFvity and social moFvaFon in voles. eLife. 10.7554/eLife.72684. PMC8594915 34726153

[44] Hung L.W., Neuner S., Polepalli J.S., Beier K.T., Wright M., Walsh J.J., Lewis E.M., Luo L., Deisseroth K., Dölen G., (2017). GaFng of social reward by oxytocin in the ventral tegmental area. Science 357, 1406–1411. 10.1126/science.aan4994. 28963257 PMC6214365

[45] Johnson Z.V., Walum H., Xiao Y., Riefkohl P.C., and Young L.J. (2017). Oxytocin receptors modulate a social salience neural network in male prairie voles. Hormones and Behavior 87, 16–24. 10.1016/j.yhbeh.2016.10.009. 27793769 PMC5207344

[46] Nardou R., Lewis E.M., Rothhaas R., Xu R., Yang A., Boyden E., and Dölen G. (2019). Oxytocin-dependent reopening of a social reward learning criFcal period with MDMA. Nature 569, 116–120. 10.1038/s41586-019-1075-9. 30944474

[47] Queen N.J., Huang W., KomaFneni S., Mansour A.G., Xiao R., Chrislip L.A., and Cao L. (2023). Social isolaFon exacerbates diet-induced obesity and peripheral inflammaFon in young male mice under thermoneutrality. iScience 26, 106259. 10.1016/j.isci.2023.106259. 36915694 PMC10006833

[48] Ding L., Bailey M.H., Porta-Pardo E., Thorsson V., Colaprico A., Bertrand D., Gibbs D.L., Weerasinghe A., Huang K., Tokheim C., (2018). PerspecFve on Oncogenic Processes at the End of the Beginning of Cancer Genomics. Cell 173, 305–320.e10. 10.1016/j.cell.2018.03.033. 29625049 PMC5916814

[49] Mousel M.R., Stroup W.W., and Nielsen M.K. (2001). Locomotor acFvity, core body temperature, and circadian rhythms in mice selected for high or low heat loss. Journal of Animal Science 79, 861–868. 10.2527/2001.794861x. 11325190

[50] Harding E.C., Franks N.P., and Wisden W. (2019). The Temperature Dependence of Sleep. FronFers in Neuroscience 13. 10.3389/fnins.2019.00336 PMC6491889 31105512

[51] Harding E.C., Franks N.P., and Wisden W. (2020). Sleep and thermoregulaFon. Current Opinion in Physiology 15, 7–13. 10.1016/j.cophys.2019.11.008. 32617439 PMC7323637

[52] McGinty D., and Szymusiak R. (1990). Keeping cool: a hypothesis about the mechanisms and funcFons of slow-wave sleep. Trends in Neurosciences 13, 480–487. 10.1016/0166-2236(90)90081-K. 1703678

[53] TaMersall G.J., Roussel D., Voituron Y., and Teulier L. (2016). Novel energy-saving strategies to mulFple stressors in birds: the ultradian regulaFon of body temperature. Proceedings of the Royal Society B: Biological Sciences 283, 20161551. 10.1098/rspb.2016.1551. PMC5046907 27655770

[54] Snyder S., and Franks P.J.S. (2016). QuanFfying the effects of sensor coaFngs on body temperature measurements. Animal Biotelemetry 4, 8. 10.1186/s40317-016-0100-0.

[55] Monteiro P., and Feng G. (2017). SHANK proteins: roles at the synapse and in auFsm spectrum disorder. Nat Rev Neurosci 18, 147–157. 10.1038/nrn.2016.183. 28179641

[56] Sheng M., and Hoogenraad C.C. (2007). The PostsynapFc Architecture of Excitatory Synapses: A More QuanFtaFve View. Annual Review of Biochemistry 76, 823–847. 10.1146/annurev.biochem.76.060805.160029. 17243894

[57] Delling J.P., and Boeckers T.M. (2021). Comparison of SHANK3 deficiency in animal models: phenotypes, treatment strategies, and translaFonal implicaFons. Journal of Neurodevelopmental Disorders 13, 55. 10.1186/s11689-021-09397-8. 34784886 PMC8594088

[58] Schön M., Lapunzina P., Nevado J., Mattina T., Gunnarsson C., Hadzsiev K., Verpelli C., Bourgeron T., Jesse S., van Ravenswaaij-Arts C.M.A., (2023). DefiniFon and clinical variability of SHANK3-related Phelan-McDermid syndrome. European Journal of Medical GeneFcs 66, 104754. 10.1016/j.ejmg.2023.104754. 37003575

[59] Uchino S., and Waga C. (2013). SHANK3 as an auFsm spectrum disorder-associated gene. Brain and Development 35, 106–110. 10.1016/j.braindev.2012.05.013. 22749736

[60] McKemy D.D. (2007). TRPM8: The Cold and Menthol Receptor. In TRP Ion Channel FuncFon in Sensory TransducFon and Cellular Signaling Cascades FronFers in Neuroscience., Liedtke W. B. and Heller S., eds. (CRC Press/Taylor & Francis). 21204488

[61] Tominaga M. (2007). NocicepFon and TRP Channels. In Transient Receptor PotenFal (TRP) Channels Handbook of Experimental Pharmacology., Flockerzi V. and Nilius B., eds. (Springer), pp. 489–505. 10.1007/978-3-540-34891-7_29.

[62] Almeida M.C., Hew-Butler T., Soriano R.N., Rao S., Wang W., Wang J., Tamayo N., Oliveira D.L., Nucci T.B., Aryal P., (2012). Pharmacological Blockade of the Cold Receptor TRPM8 AMenuates Autonomic and Behavioral Cold Defenses and Decreases Deep Body Temperature. J. Neurosci. 32, 2086–2099. 10.1523/JNEUROSCI.5606-11.2012. 22323721 PMC3566779

[63] BauFsta D.M., Siemens J., Glazer J.M., Tsuruda P.R., Basbaum A.I., Stucky C.L., Jordt S.-E., and Julius D. (2007). The menthol receptor TRPM8 is the principal detector of environmental cold. Nature 448, 204–208. 10.1038/nature05910. 17538622

[64] Reimúndez A., Fernández-Peña C., García G., Fernández R., Ordás P., Gallego R., Pardo-Vazquez J.L., Arce V., Viana F., and Señarís R. (2018). DeleFon of the Cold Thermoreceptor TRPM8 Increases Heat Loss and Food Intake Leading to Reduced Body Temperature and Obesity in Mice. J. Neurosci. 38, 3643–3656. 10.1523/JNEUROSCI.3002-17.2018. 29530988 PMC6705917

[65] Caterina M.J., Leffler A., Malmberg A.B., MarFn W.J., Trapon J., Petersen-Zeitz K.R., Koltzenburg M., Basbaum A.I., and Julius D. (2000). Impaired nocicepFon and pain sensaFon in mice lacking the capsaicin receptor. Science 288, 306–313. 10.1126/science.288.5464.306. 10764638

[66] Alberts J.R. (2007). Huddling by rat pups: ontogeny of individual and group behavior. Dev Psychobiol 49, 22–32. 10.1002/dev.20190. 17186514

[67] Morrison I. (2016). Keep Calm and Cuddle on: Social Touch as a Stress Buffer. AdapFve Human Behavior and Physiology 2, 344–362. 10.1007/s40750-016-0052-x.

[68] Wills G.D., Wesley A.L., Moore F.R., and Sisemore D.A. (1983). Social interacFons among rodent conspecifics: A review of experimental paradigms. Neuroscience & Biobehavioral Reviews 7, 315–323. 10.1016/0149-7634(83)90035-0. 6366644

[69] Villegas M., Bozinovic F., and Sabat P. (2013). Interplay between group size, huddling behavior and basal metabolism: an experimental approach in the social degus. Journal of Experimental Biology, jeb.096164. 10.1242/jeb.096164. 24311802

[70] Nakamura K., and Morrison S.F. (2022). Central sympatheFc network for thermoregulatory responses to psychological stress. Autonomic Neuroscience 237, 102918. 10.1016/j.autneu.2021.102918. 34823147

[71] Kataoka N., Shima Y., Nakajima K., and Nakamura K. (2020). A central master driver of psychosocial stress responses in the rat. Science 367, 1105–1112. 10.1126/science.aaz4639. 32139538

[72] Kataoka N., Hioki H., Kaneko T., and Nakamura K. (2014). Psychological Stress AcFvates a Dorsomedial Hypothalamus-Medullary Raphe Circuit Driving Brown Adipose Tissue Thermogenesis and Hyperthermia. Cell Metabolism 20, 346–358. 10.1016/j.cmet.2014.05.018. 24981837

[73] Oka T. (2015). Psychogenic fever: how psychological stress affects body temperature in the clinical populaFon. Temperature 2, 368–378. 10.1080/23328940.2015.1056907. PMC4843908 27227051

[74] Da Prato L.C., Zayan U., Abdallah D., Point V., Schaller F., Pallesi-Pocachard E., Montheil A., Canaan S., Gaiarsa J.-L., Muscatelli F., (2022). Early life oxytocin treatment improves thermo-sensory reacFvity and maternal behavior in neonates lacking the auFsm-associated gene Magel2. Neuropsychopharmacol. 47, 1901–1912. 10.1038/s41386-022-01313-5. PMC9485246 35396500

[75] Spain D., Sin J., Linder K.B., McMahon J., and Happé F. (2018). Social anxiety in auFsm spectrum disorder: A systemaFc review. Research in AuFsm Spectrum Disorders 52, 51–68. 10.1016/j.rasd.2018.04.007.

[76] Maddox B.B., and White S.W. (2015). Comorbid Social Anxiety Disorder in Adults with AuFsm Spectrum Disorder. J AuFsm Dev Disord 45, 3949–3960. 10.1007/s10803-015-2531-5. 26243138

[77] Spain D., Happé F., Johnston P., Campbell M., Sin J., Daly E., Ecker C., Anson M., Chaplin E., Glaser K., (2016). Social anxiety in adult males with auFsm spectrum disorders. Research in AuFsm Spectrum Disorders 32, 13–23. 10.1016/j.rasd.2016.08.002.

[78] Harshaw C., Leffel J.K., and Alberts J.R. (2018). Oxytocin and the warm outer glow: Thermoregulatory deficits cause huddling abnormaliFes in oxytocin-deficient mouse pups. Hormones and Behavior 98, 145–158. 10.1016/j.yhbeh.2017.12.007. 29277701 PMC5828998

[79] Lee C.R., Chen A., and Tye K.M. (2021). The neural circuitry of social homeostasis: Consequences of acute versus chronic social isolaFon. Cell 184, 1500–1516. 10.1016/j.cell.2021.02.028. 33691140 PMC8580010

